# Dance activity interventions targeting cognitive functioning in older adults with mild cognitive impairment: A meta-analysis

**DOI:** 10.3389/fpsyg.2022.966675

**Published:** 2022-09-27

**Authors:** Yuxin Yuan, Xiaofen Li, Wanxu Liu

**Affiliations:** School of Art, Beijing Sport University, Beijing, China

**Keywords:** dance activity, dance exercise, MCI, cognitive function, meta-analysis

## Abstract

**Objectives:**

To comprehensively determine the effect of dance activities on the cognitive functions and its sub-domains of older adults with mild cognitive impairment (MCI).

**Methods:**

We obtained data from PubMed, Web of Science, EBSCO, China national knowledge infrastructure, Wanfang data, and VIP databases from 2017/01/01 to 2022/03/01. We included trials of older adults with MCI that underwent dance activity intervention and fulfilled the inclusion criteria. Two researchers independently assessed the quality of the study using the Cochrane risk of the bias assessment tool. Meta-analysis was performed when data were available, with further subgroup analysis, using Review Manager 5.4, and sensitivity analysis was performed using Stata software 15.1.

**Results:**

Search terms yielded 183 articles, of which 12 fulfilled the inclusion criteria. This included 7 high-quality studies and 5 medium-quality studies. A total of 820 older adults were analyzed. Results showed that dance activity had beneficial effects for global cognition [SMD_MMSE_ = 0.65, 95% CI_MMSE_ (0.20, 1.09), *p*_MMSE_ = 0.004; SMD_MoCA_ = 0.87, 95% CI_MoCA_ (0.44, 1.29), *p*_MoCA_ < 0.0001], memory [SMD = 0.61, 95% CI (0.35, 0.88), *p* < 0.00001], visuospatial function [SMD = −0.39, 95% CI (−0.60, −0.19), *p* = 0.0002], cognitive flexibility [SMD = −0.31, 95% CI (−0.52, −0.11), *p* = 0.003], attention [SMD = 0.34, 95% CI (0.07, 0.61), *p* = 0.01], and balance [SMD = 1.25, 95% CI (0.06, 2.44), *p* = 0.04]. Further subgroup analysis showed that open-skill dance activity (OSDA) was more effective in promoting global cognition in older adults with MCI than closed-skill dance activity (CSDA) because of the different stimulation provided by the two types of dance activities in the brain regions of the older adults (*p* = 0.0002). It could be speculated that dance activity improved cognitive function mainly by affecting the microstructure and function of the cingulate tract, hippocampus, cardiovascular function, and other brain areas of older adults with MCI.

**Conclusion:**

Dance activities can significantly improve global cognition, memory, visuospatial function, cognitive flexibility, attention, and balance in older adults with MCI. However, more trials with rigorous study designs are necessary to provide more concrete evidence in the future.

## Introduction

The rapid aging of the global population entails an increase in the prevalence of cognitive impairment in the older generation. A previous study reported that in the past decade, the prevalence of mild cognitive impairment (MCI) in the elderly population of China was 19%, which was higher than that of Japan during the same period (Shi et al., 2022). MCI is an intermediate condition between normal aging and Alzheimer's Disease (AD), which has a strong early warning effect on AD (Zhao et al., 2018). MCI is mainly manifested as the decline of brain function, memorial function, and executive function (Yang et al., 2022). MCI can easily develop into severe cognitive impairment and irreversible functional impairment (Wang and Ma, 2021). Therefore, early intervention is crucial to inhibit the transformation of MCI to AD.

Pharmacological and non-pharmacological therapies are the mainstays of treatment to preserve the cognitive function of older adults with MCI. However, because of the uncertain therapeutic effects and the risks of side effects of pharmacological treatment (Hu et al., 2017; Edmonds et al., 2018; Petersen et al., 2018), non-pharmacological therapies are now advocated to delay MCI-related decline. Previous studies have reported the benefits of non-pharmacological interventions, such as aerobic exercise, physical activity, and gymnastics, to prevent the decline of brain function by improving cognitive function and its sub-domains *via* the increase of cerebral blood flow (Alfini et al., 2019; Wang et al., 2019).

In recent years, dance has become a popular physical activity in the elderly because it integrates several aspects, such as audio-visual perception, physical perception, and emotional expression (Pereira et al., 2019). Because it is a non-pharmacological cognitive means of intervention, it is beneficial to the episodic memory, executive function, and global cognition of older adults (Fang et al., 2017). Compared with other non-pharmacological interventions, such as computer-assisted training, dance activities have lesser requirements for venues and equipment and are relatively more convenient to implement. Dance activities have become increasingly accepted. Dance activity intervention has been reported to have positive effects on cognitive function, including executive function, balance, verbal recognition memory, and visual delayed recall in older adults with MCI (Lazarou et al., 2017; Qi et al., 2019; Bisbe et al., 2020; Wang et al., 2020). However, there is still a lack of evidence to find out the most effective type and dose of dance activity intervention. Therefore, the purpose of this study was to conduct a meta-analysis to describe and determine the effect of dance activity intervention on global cognitive function and its subdomains in older adults with MCI, as well as a subgroup analysis to determine the influencing factors.

## Methods

The selection procedure, study identification, and critical appraisal of the research studies were conducted according to the checklist presented in the Preferred Reporting Items for Systematic Reviews and Meta-analyses (PRISMA) statement (Page et al., 2021).

### Search strategy

We performed a comprehensive search in the following six databases: PubMed, Web of Science, EBSCO, China National Knowledge Infrastructure, WanfangData, and VIP Data. We searched the databases from January 1, 2017, to March 1, 2022. We used the PICOS framework to identify the keywords and also used the Boolean search method. Taking Web of Science as an example, the search formula was as follows: [(MCI OR mild cognitive impairment) AND (danc^*^ OR dance therapy) AND (cogniti^*^ OR cogniti^*^ function OR memor^*^ OR executive function OR special function OR visual function OR attention)].

### Inclusion and exclusion criteria

Trials examining the effect of dance activities on cognitive function and its sub-domains in older adults with MCI were selected following PICOS (Participants, Intervention, Comparison, Outcomes, and Study design) inclusion criteria: (1) participants aged 50~85 years old of any sex, with a diagnosis of MCI, with a history of memory impairment lasting ≥3 months; (2) intervention with dance activities, dance exercises, or dance therapy, without restriction regarding the control method; (3) comparison with any type of control means; (4) outcome with at least one validated quantitative generic rating scale of global cognition or its sub-domain; (5) controlled designs, both parallel groups or crossover, with or without randomization or blinding.

The studies on older adults suffering from other mental diseases were excluded. Review articles, retrospective studies, case reports or series, protocols, editorials, notes, and commentaries were not included. Only articles with full data access and written in English or Chinese were considered eligible for inclusion.

### Study selection

Two researchers (YY and LW) independently screened all the included studies by reading the title, abstract, and, if necessary, the main text of the article to determine whether the studies were eligible for review. The reasons for the ineligibility of a study were recorded. Subsequently, the two researchers had a discussion to reach a consensus. The third researcher (LX) intervened in the discussion in the case of a disagreement. Any discrepancies between the researchers were resolved by discussion.

### Methodological quality appraisal

The Cochrane Collaboration Risk of Bias tool was used to assess the quality of the included studies. Based on the Cochrane Handbook, the evaluation included random sequence generation, allocation concealment, blinding of outcome assessment, incomplete outcome data, selective reporting, and other biases. All aspects of the included studies were assessed as low, unclear, or high risk of bias (Higgins et al., 2019).

### Data extraction and synthesis

All the relevant information about each study was extracted using a self-designed standardized form, which included basic information (the first author, the year of publication, and the region) and experimental information (such as participants, intervention characteristics, and outcome measures). Data missing was handled by contacting the authors of the included studies. Any discrepancy was resolved by discussion.

Meta-analysis was performed using Review Manager 5.4, and a leave-one-out analysis was performed to assess the sensitivity using Stata software 15.1. The summary statistics for each outcome were the mean change from baseline and standard deviations (SD) of the mean change. The mean change in each group was obtained by subtracting the final mean from the baseline mean. The SD of mean change was computed in line with Follmann et al. (1992), which assumed a conservative correlation coefficient of 0.5. The standardized mean difference (SMD) and 95% confidence interval (CI) were calculated for the summary effect of continuous data. Results were considered significant when the CI did not include zero.

Heterogeneity was determined by the Cochrane Q statistic and the *I*^2^ statistic. Cochrane Q statistic was used to test the heterogeneity, and the *I*^2^ statistic was used to evaluate its value. The value of the *I*^2^ statistic of 25, 50, and 75% indicated that the degree of heterogeneity between studies is low, moderate, and high (Higgins et al., 2003). The fixed-effects model was used when the *I*^2^ < 50% and the random-effects model was used when the *I*^2^> 50%. The studies with a greater variance in their effect size estimate contributed less to the summary effect. When high heterogeneity exists, we conducted subgroup analysis based on several variables. We used Egger's regression asymmetry test to test the publication bias (Egger et al., 1997). We conducted a leave-one-out analysis to observe the stability of the results.

## Results

### Search process

**Figure 1:**
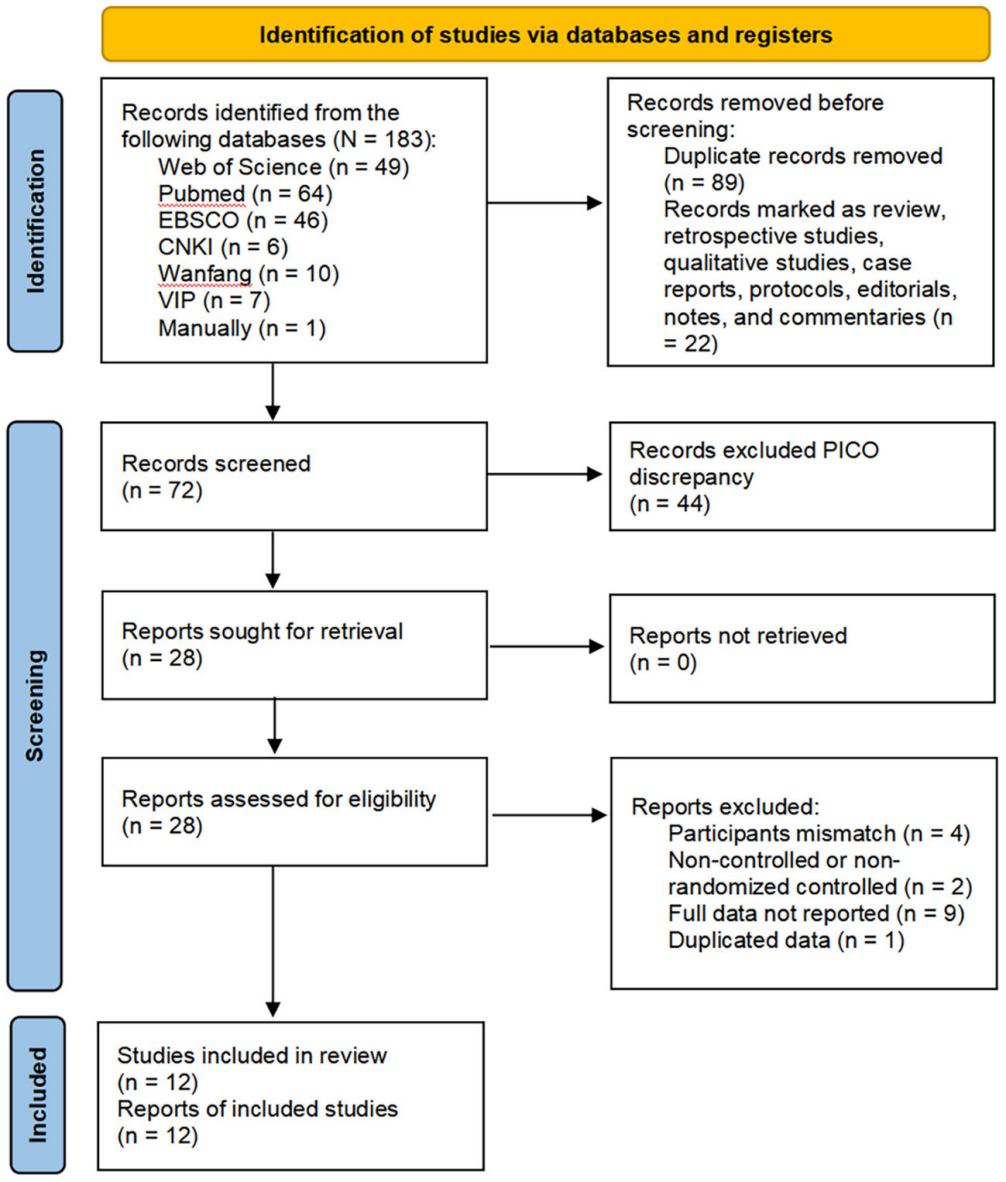
Literature selection flow diagram.

The results of the search process are shown in Figure 1. The bibliographical search yielded 183 citations, including 1 citation searched manually, published between 2017 and March 2022. After reading the title and abstract, 155 citations were excluded due to duplication and non-interventional studies. The full text of the remaining studies (*n* = 28) was assessed for eligibility based on the inclusion criteria. A total of 12 studies were considered eligible for review.

### Risk of bias assessment

**Figure 2:**
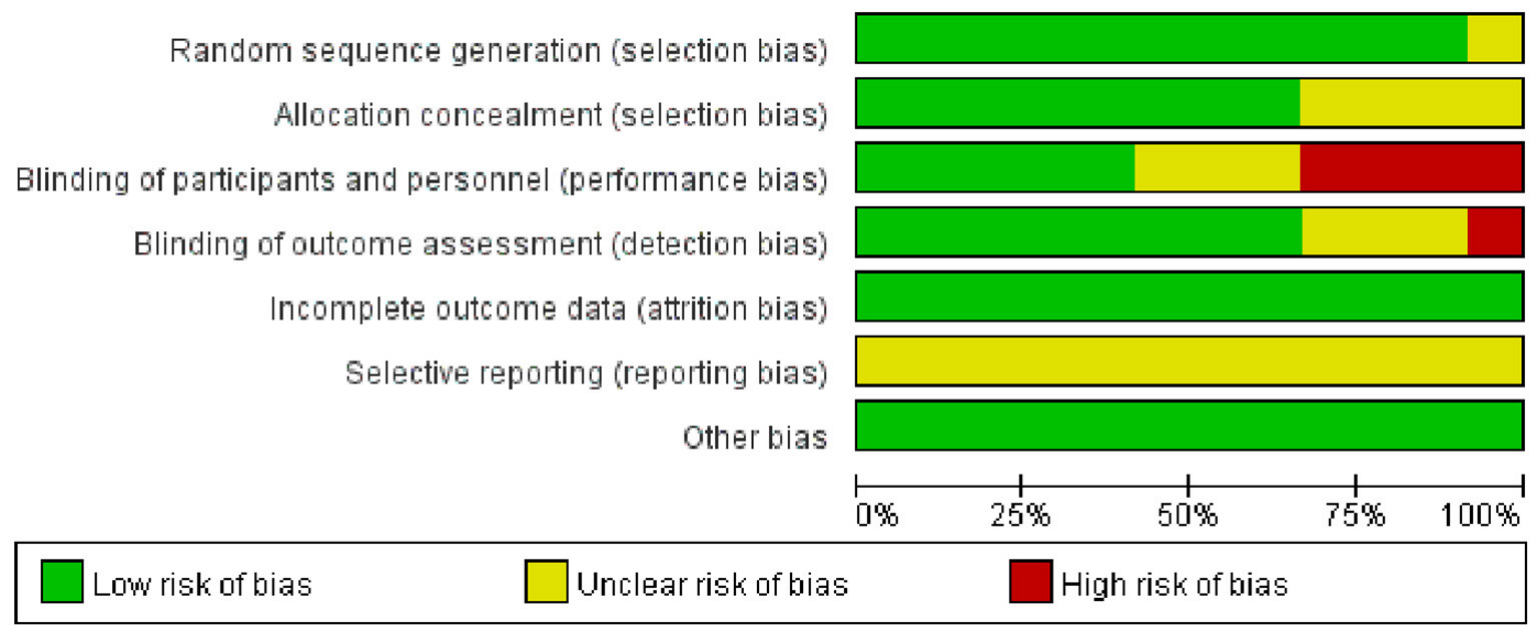
Percentage of biased items included.

**Figure 3:**
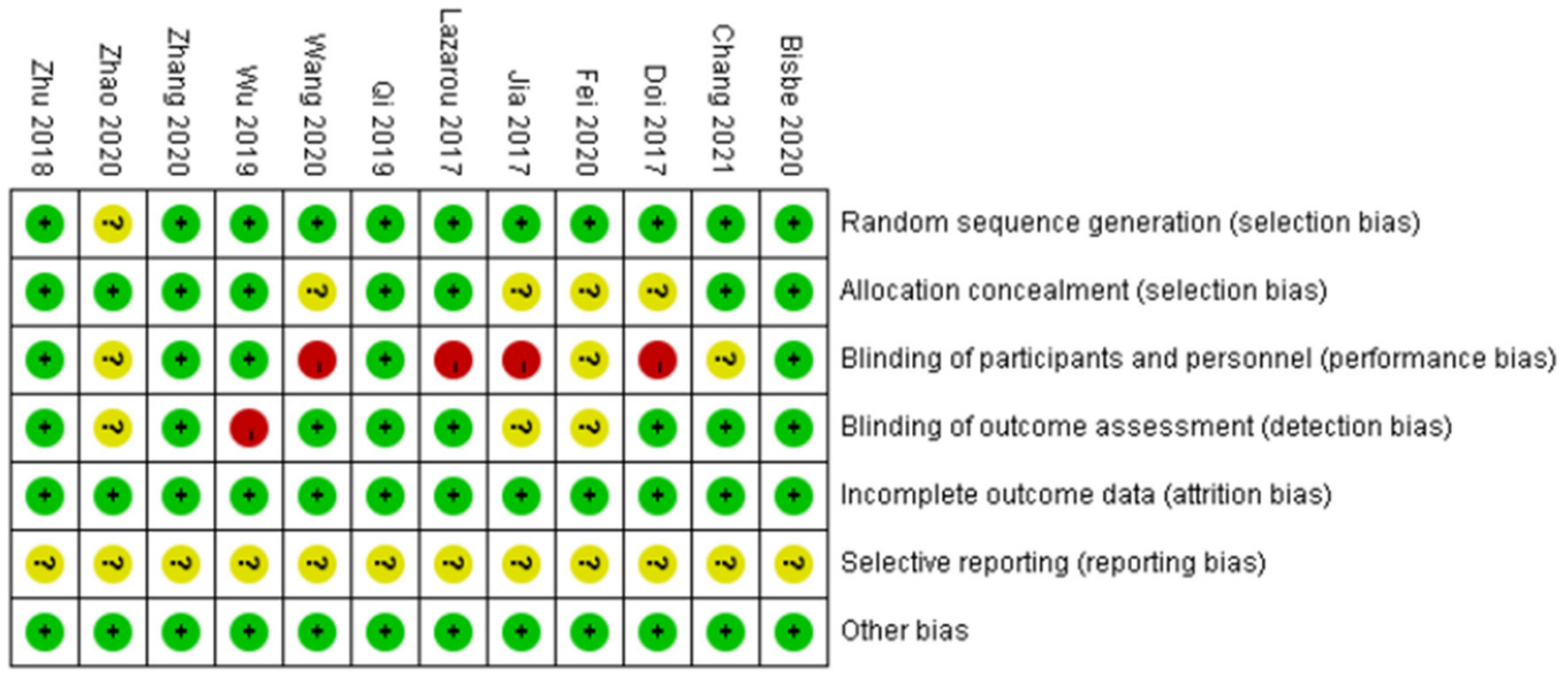
Risk of bias assessment results of included studies.

We performed a Cochrane risk of bias assessment for each study. The full results are shown in Figures 2, 3. Overall, the included studies had good methodological quality, with seven studies rated with strong quality and five studies with moderate quality.

### Study characteristics

The characteristics of the study are shown in Tables 1, 2. The included studies were performed in China (*n* = 9) (Jia, 2017; Zhu et al., 2018; Qi et al., 2019; Wu et al., 2019; Fei and Cai, 2020; Wang et al., 2020; Zhang et al., 2020; Zhao and Li, 2020; Chang et al., 2021), Spain (*n* = 1) (Bisbe et al., 2020), Japan (*n* = 1) (Doi et al., 2017), and Greece (*n* = 1) (Lazarou et al., 2017). A total of 820 participants were covered in this review, of which 414 were included in the intervention groups and 406 in the control groups. The sample size of a single study ranged from 31 to 129. All the participants were adult older adults with MCI, aged between 50 and 85 years. Six studies reported the percentage of participants' gender, while 2 studies only reported at baseline, and 4 studies did not report it. The interventions were classified into the following four categories: (1) aerobic dance (*n* = 4), (2) square dance (*n* = 4), (3) ballroom dance (*n* = 3), and choreographed exercise (*n* = 1). Because of the high popularity and acceptance of square dance and aerobic dance in China, research studies in Chinese used these types of dance activities performed by a single person as intervention, whereas studies from Spain, Japan, and Greece used dance activities with strong social characteristics such as ballroom dance and choreographed exercise. The majority of the intervention intensity was light-to-moderate (*n* = 9). The intervention was for 30–60 min, 1–3 times per week, and lasted for 9–40 weeks.

**Table 1:** General characteristics of the included studies.

**Study**	**Sample size (male%)**	**Age**	**Study design**	**Intervention**	**Motor skill**	**Intensity**	**Control condition**	**Time (min)**	**Frequency (times/week)**	**Intervention length (week)**
Bisbe et al. (2020) Spain	I = 17 (/) C = 14 (/)	74.9	RCT	Choreographed exercise	OS	Light to moderate	Physical therapy	60	2	12
Chang et al. (2021) China	I = 62 (/) C = 47 (/)	76.3	RCT	Square dance	CS	HR 100–140 beats per min	Usual practice	30	3	18
Doi et al. (2017) Japan	I = 55 (49.3) C = 63 (53.7)	75.9	RCT	Ballroom dance	OS	Not mentioned	Health education	60	1	40
Lazarou et al. (2017) Greece	I = 66 (/) C = 63 (/)	66.9	RCT	Ballroom dance	OS	Not mentioned	Blank control	60	2	40
Qi et al. (2019) China	I = 16 (31.3) C = 16 (25.0)	69.9	RCT	Aerobic dance	CS	60–80% HRmax	Usual care	35	3	12
Wang et al. (2020) China	I = 33 (21.2) C = 33 (36.4)	81	CT	Square dance	CS	Not mentioned	Usual lifestyle	40	3	12
Zhu et al. (2018) China	I = 27 (-) C = 31 (-)	72	RCT	Aerobic dance and regular care	CS	60–80% HRmax	Regular care	35	3	12
Fei and Cai (2020) China	I = 41 (/) C = 41 (/)	65~85	RCT	Ballroom dance	OS	60–70% HRmax	Health education	60	3	24
Wu et al. (2019) China	I = 27 (-) C = 27 (-)	69.6	RCT	Aerobic dance	CS	60% HRmax	Health education	35	3	12
Zhang et al. (2020) China	I = 16 (31.3) C = 16 (25.0)	69.9	RCT	Aerobic dance	CS	60% HRmax	Health education	35	3	12
Zhao and Li (2020) China	I = 31 (16.1) C = 32(18.8)	72.3	CT	Square dance and health education	CS	60–70% HRmax	Health education	60	3	12
Jia (2017) China	I = 23 (0) C = 23(0)	64.5	RCT	Square dance and medicine	CS	Light to moderate	Medicine	40–60	1	9

I, intervention group; C, control group; RCT, Random controlled trial;/, not reported;-, not available; OS, open-skill; CS, closed-skill.

**Table 2:** Summary of outcome measures.

**Outcome**	**Measurement tool**	**Study**
Global cognition	MMSE, Mini-mental state examination	Bisbe, Doi, Lazarou, Wang, Fei, Wu, Zhang, Jia
	MoCA, Montreal cognitive assessment	Chang, Lazarou, Wang, Zhu, Fei, Wu, Zhang, Zhao
Visuospatial function	TMT-A, Trail making test-A	Bisbe, Doi, Zhu, Fei, Wu, Zhang
Cognitive flexibility	TMT-B, Trail making test-B	Bisbe, Doi, Zhu, Fei, Wu, Zhang
Memory	DST, Digit span test	Qi, Zhu
	LM, Logical memory	Zhu, Wu, Zhang
Attention	TEA, Test of everyday attention	Lazarou
	SDMT, Symbol digit modalities test	Qi, Zhu
Balance	BBS, Berg balance scale	Bisbe, Chang, Qi, Wang, Fei, Wu

### The overall analysis of the effects of dance intervention

#### Primary outcome: Global cognition

##### MMSE

**Figure 4:**
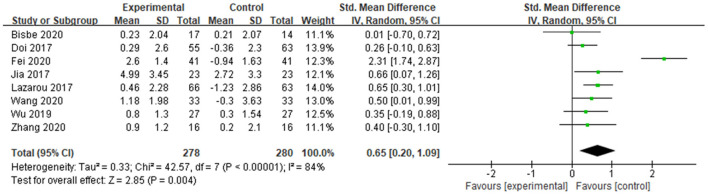
Effects of dance activities on global cognition (MMSE).

A total of 8 studies determined the effects of dance activities on global cognition by mini-Mental State Examination (MMSE). The score of MMSE is positively correlated with the cognitive functions of the subject, and the weight of each study was determined (Figure 4). The result showed significant differences between groups [SMD = 0.65, 95% CI (0.20, 1.09), *p* = 0.004], with high and significant heterogeneity (*I*^2^ = 84% and Chi^2^ *p* < 0.00001) (Figure 4).

##### MoCA

**Figure 5:**
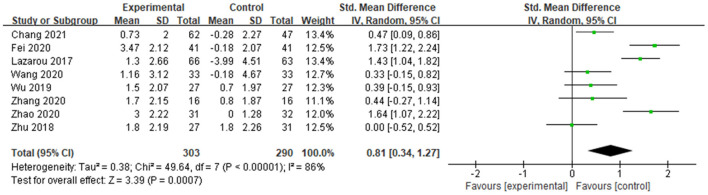
Effects of dance activities on global cognition (MoCA).

A total of 8 studies used Montreal cognitive assessment (MoCA) to determine the effects of dance activities on global cognition. The score of MoCA shows a positive correlation with the cognitive functions of the subjects, and the weight of each study was determined (Figure 5). The meta-analysis showed significant differences between groups [SMD = 0.87, 95% CI (0.44, 1.29), *p* < 0.0001], with high and significant heterogeneity (*I*^2^ = 83% and Chi^2^ *p* < 0.00001) (Figure 5).

The results of the above indicate that dance activity could enhance the global cognition of adult older adults with MCI.

#### Secondary outcome: Cognitive sub-domains

Apart from the effects on global cognition, a majority of studies also determined the effects of dance activity on specific cognitive sub-domains such as memory, visuospatial function, cognitive flexibility, attention, and balance. Some subdomains of cognitive function were reported with multiple measures. In these circumstances, when calculating the mean effect size estimate, we averaged the effect sizes across the measures and components.

##### Memory

A total of 5 studies determined the effects of dance activities on memory, of which 2 studies used the digit span test (DST), and 3 studies used logical memory (LM). The scores of both DST and LM were positively correlated with the memorial functions of the subjects. The meta-analysis showed significant differences between groups in memory [SMD = 0.61, 95% CI (0.35, 0.88), *p* < 0.00001], with low heterogeneity (*I*^2^ = 4% and Chi^2^ *p* = 0.39), which indicates dance activity positively affects the memorial function of older adults with MCI.

##### Visuospatial function

A total of 6 studies conducted the trail-making test-A (TMT-A). The score of the TMT-A test was negatively correlated with the visuospatial functions of the subjects. The meta-analysis showed significant differences between groups in visuospatial function [SMD = −0.39, 95% CI (−0.61, −0.19), *p* = 0.003], with moderate heterogeneity (*I*^2^ = 50% and Chi^2^ *p* = 0.08), which indicates that the visuospatial function was improved after dance intervention.

##### Cognitive flexibility

A total of 6 studies examined the results of the trail-making test-B (TMT-B). The score of the TMT-B test was negatively correlated with the cognitive flexibility of the subjects. The meta-analysis showed significant differences between the groups in terms of cognitive flexibility [SMD = −0.31, 95% CI (−0.52, −0.11), *p* = 0.0002], with homogeneity (*I*^2^ = 0%, Chi^2^ *p* = 0.45), which indicates that dance activity intervention improved participants' cognitive function.yy

##### Attention

A total of 3 studies examined the effects of dance activities on attention, of which 1 study used the test of everyday attention (TEA), and 2 studies used the symbol digit modalities test (SDMT). The scores of both TEA and SDMT were positively correlated with the attention of the subjects. The meta-analysis showed significant differences between groups in attention [SMD = 0.34, 95% CI (0.07, 0.61), *p* = 0.01], with homogeneity (*I*^2^ = 0% and Chi^2^ *p* = 0.95), which indicates that participants' attention could be enhanced by dance activity intervention.

##### Balance

A total of 6 studies determined the effects of dance activities on balance. The score of the Berg balance scale (BBS) test was positively correlated with the balance of the subjects. The meta-analysis showed significant differences between groups in cognitive flexibility [SMD = 1.25, 95% CI (0.06, 2.44), *p* = 0.04], with high and significant heterogeneity (*I*^2^ = 96%, Chi^2^ *p* < 0.00001), which indicates that the balance of participants was enhanced after dance intervention (Table 3).

**Table 3:** Summary of the effects of dance activities on cognitive sub-domains.

**Outcome measures**	**Study**	***SMD* (95%CI)**	***p***	***I^2^***
Global cognition	11	0.73 [0.41, 1.04]	< 0.00001	84%
MMSE	8	0.65 [0.20, 1.09]	< 0.00001	84%
MoCA	8	0.87 [0.44, 1.29]	< 0.00001	83%
Memory	4	0.61 [0.35, 0.88]	< 0.00001	4%
DST	2	0.42 [−0.00, 0.84]	0.05	0%
LM	3	0.74 [0.40, 1.08]	< 0.0001	25%
Visuospatial function	6	−0.39 [−0.60, −0.19]	0.0002	50%
TMT-A	6	−0.39 [−0.60, −0.19]	0.0002	50%
Cognitive flexibility	6	−0.31 [−0.52, −0.11]	0.003	0%
TMT-B	6	−0.31 [−0.52, −0.11]	0.003	0%
Attention	3	0.34 [0.07, 0.61]	0.01	0%
TEA	1	0.37 [0.02, 0.72]	0.04	-
SDMT	2	0.29 [−0.13, 0.71]	0.17	0%
Balance	6	1.25 [0.06, 2.44]	0.04	96%
BBS	6	1.25 [0.06, 2.44]	0.04	96%

### Subgroup analysis

#### The effects of different dance modalities on global cognition

**Figure 6:**
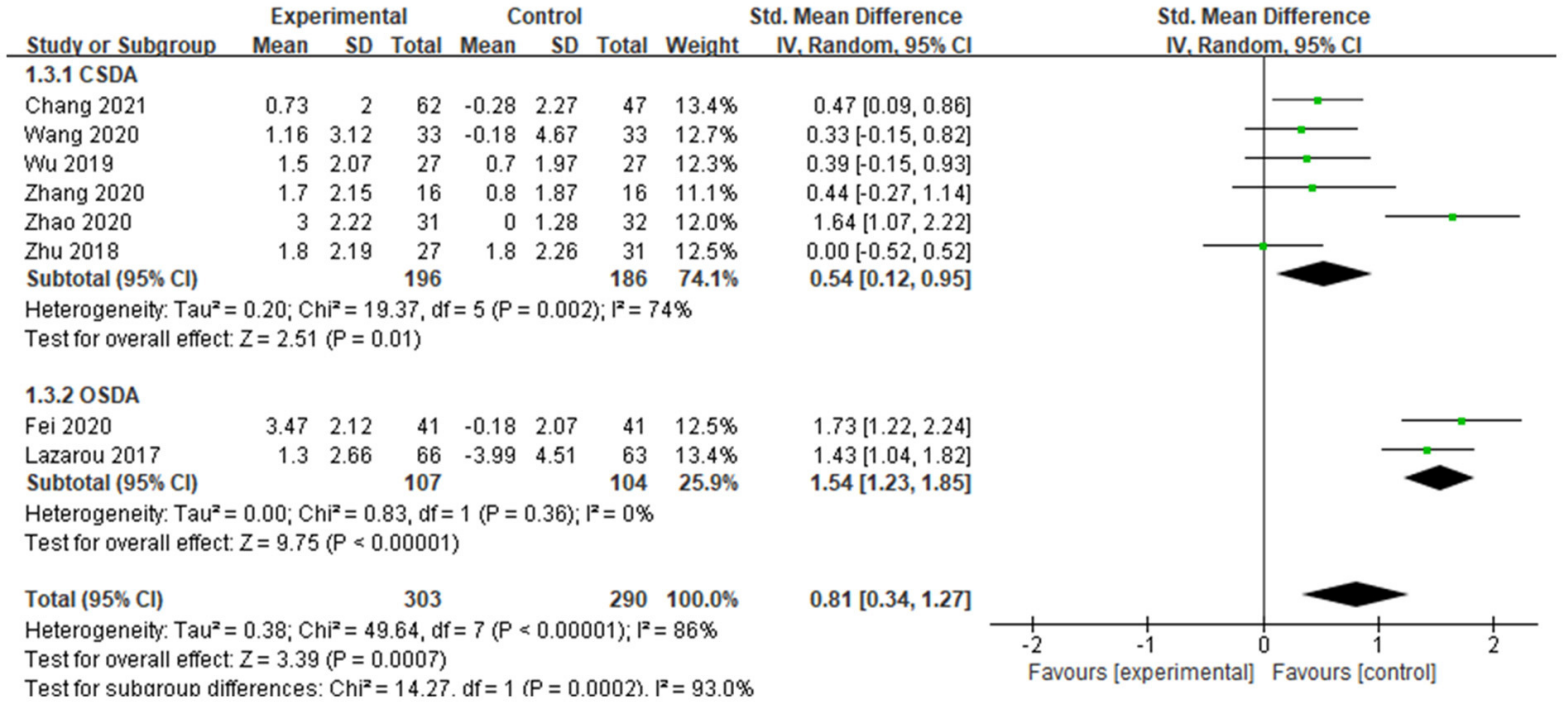
Effects of different dance motor skills on global cognition.

Figure 6 and Table 4 show the effects of different dance modalities based on the motor skills of the interventions on global cognition. A total of 6 studies reported the effects of closed-skill dance activity (CSDA), specifically aerobic dance and square dance, and 2 studies reported the effects of open-skill dance activity (OSDA) and specifically aerobic ballroom dance. Compared with the control group, a significant effect was found in CSDA on global cognition [SMD = 0.61, 95% CI (0.24, 0.98), *p* = 0.001], with high heterogeneity (*I*^2^ = 67% and Chi^2^ *p* = 0.010), whereas a large, significant effect was found in OSDA [SMD = 1.54, 95% CI (1.23, 1.85), *p* < 0.00001], with homogeneity (*I*^2^ = 0% and Chi^2^ *p* = 0.36).

**Table 4:** Effects of different dance motor skills on global cognition.

**Dance motor skills**	**Study**	**Heterogeneity**		**Test for overall effect**		***SMD* [95% CI]**	**Difference between groups *p***
		***p***	***I^2^***	***Z***	***p***		
Closed	6	0.010	67%	3.24	0.001	0.61 [0.24, 0.98]	0.0002*
Open	2	0.36	0%	9.75	< 0.00001	1.54 [1.23, 1.85]	

*The difference was significant.

#### The effects of different intervention lengths and frequencies on global cognition

Table 5 shows the effects of different dance interventions based on the length and frequencies of the dance intervention on global cognition. We used a median split to determine a cutoff value of intervention length (12 weeks) and intervention frequency (3 times per week). In terms of intervention length, no difference was found between the intervention of more than 12 weeks [SMD = 1.20, 95% CI (0.45, 1.95), *p* = 0.002] and the intervention of 12 weeks or fewer than 12 weeks [SMD = 0.55, 95% CI (0.01, 1.10), *p* = 0.05] (*p* = 0.17). In terms of frequency, the result showed a significant effect on both 1–2 times per week [SMD = 1.43, 95% CI (1.04, 1.82), *p* < 0.00001] and 3 times per week [SMD = 0.71, 95% CI (0.22, 1.20), *p* = 0.005], and the difference between two groups was significant (*p* = 0.03).

**Table 5:** Effects of different intervention lengths and frequencies on global cognition (MMSE).

**Subgroups**		**Study**	**Heterogeneity**		**Test for overall effect**		***SMD* [95% CI]**	**Difference between groups *p***
			***p***	***I^**2**^***	***Z***	***p***		
Length (weeks)	≤ 12	5	0.72	0%	3.08	0.002	0.41 [0.15, 0.68]	0.24
	>12	3	< 0.00001	95%	2.01	0.04	1.05 [0.03, 2.07]	
Frequency (times per week)	1–2	4	0.24	29%	3.11	0.002	0.44 [0.16, 0.71]	0.36
	3	4	< 0.00001	91%	1.88	0.06	0.89 [−0.04, 1.82]	

#### The effects of dance activity intervention on global cognition of different age groups

The effects of dance intervention on global cognition of different age groups are shown in Table 6. We divided the participants into two age groups using a median split (group 1: ≤ 70 years old and group 2:>70 years old). There was a significant effect in both groups [SMD_MMSE1_ = 0.56, 95% CI (0.31, 0.80), *p* < 0.0001; SMD_MMSE2_ = 0.30, 95% CI (0.03, 0.57), *p* = 0.03; SMD_MoCA1_ = 0.78, 95% CI (0.03, 1.54), *p* = 0.0001; SMD_MoCA2_ = 0.71, 95% CI (0.18, 1.24), *p* < 0.00001]. There was no significant difference between the two age groups (*p*_*MMSE*_ = 0.29, *p*_*MoCA*_ = 0.87).

**Table 6:** The effects of different age groups of subjects on global cognition.

**Age groups**		**Study**	**Heterogeneity**		**Test for overall effect**		***SMD* [95% CI]**	**Difference between groups *p***
			***p***	***I^2^***	***Z***	***p***		
MMSE	Group 1: ≤ 70	4	0.76	0%	4.40	< 0.0001	0.56 [0.31, 0.80]	0.29
	Group 2: >70	3	0.52	0%	2.17	0.03	0.30 [0.03, 0.57]	
MoCA	Group 1: ≤ 70	3	0.007	66%	3.80	0.0001	0.78 [0.03, 1.54]	0.87
	Group 2: >70	4	0.03	55%	4.52	< 0.00001	0.71 [0.18, 1.24]	

### Sensitivity analysis

**Figure 7:**
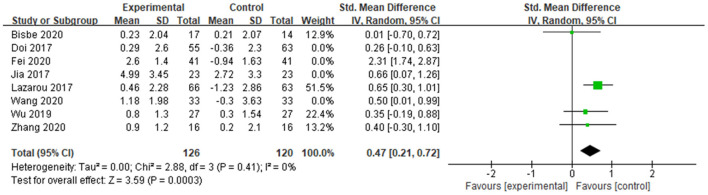
Result of studies with strong quality (MMSE).

**Figure 8:**
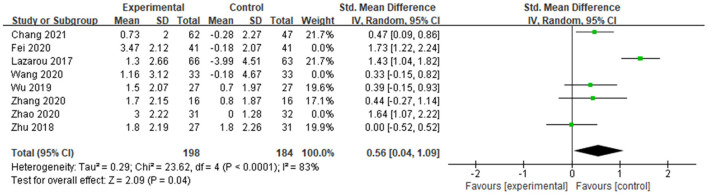
Result of studies with strong quality (MoCA).

A sensitivity analysis was performed, which excluded 5 poor-quality studies, for global cognition, with the effect of MMSE and MoCA both slightly decreased (MMSE decreased from 0.65 to 0.47, whereas MoCA decreased from 0.87 to 0.66). The result indicated the potential publication bias in the excluded studies (Figures 7, 8).

We conducted a sensitivity analysis examining a correlation of 0 or 0.9. The results showed that both effects of MMSE and MoCA remained significant (When the correlation were 0, 0.5, and 0.9, SMD_MMSE_ were 0.44, 0.66, and 0.96, SMD_MoCA_ were 0.66, 0.87, and 1.69, respectively).

**Figure 9:**
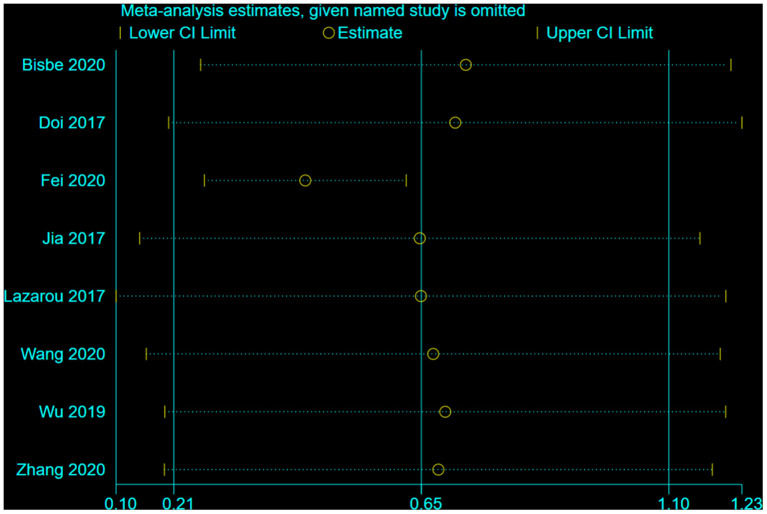
The influence of each study on the outcome of the meta-analysis (MMSE).

**Figure 10:**
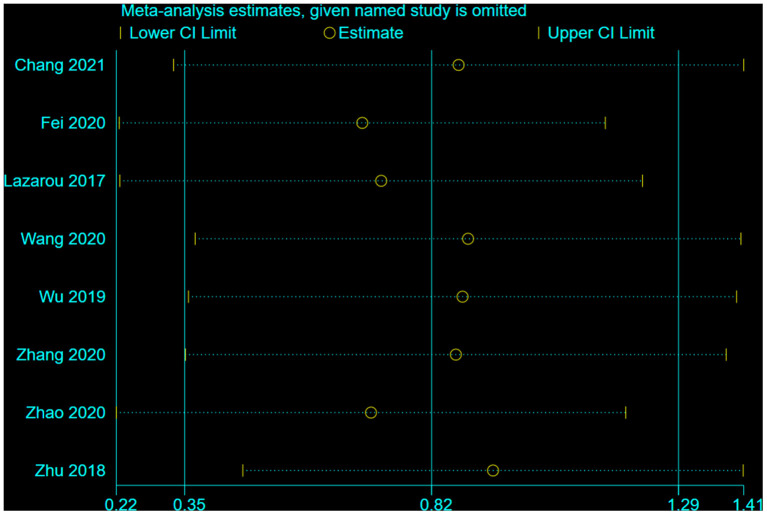
The influence of each study on the outcome of the meta-analysis (MoCA).

We further conducted a leave-one-out analysis to evaluate the influence of each study on the outcome of meta-analysis (Figures 9, 10). The results showed that after removing a study, the effect size remained significant. All the results above indicated that the included literature's stability was acceptable, and no specific study was significantly influential in the effect.

### Publication bias

We used Egger's test to assess the publication bias of the studies. The *P*-values were 0.755 (MMSE) and 0.818 (MoCA), implying no significant publication bias in this meta-analysis.

## Discussion

### Summary of findings

In the present meta-analysis study, we determined the effects of dance activities on the cognitive functions of older adults with MCI and further explored the variations when the intervention characteristics were different through the trials published in the last 5 years, which indicates the novelty of the data and the conclusion. Overall, there was a clear trend that dance intervention positively impacted global cognition and its sub-domains, including memory, visuospatial function, cognitive flexibility, attention, and balance. Our findings were in accordance with those of a recent meta-analysis (Wu et al., 2021). Memory problems are one of the most common signs of older adults with MCI (Chan et al., 2020), and encouragingly, previous studies have proved that dance was shown to be useful in increasing global cognition, memory, and learning in aging population and individuals with MCI (Porat et al., 2016; Wang et al., 2018), and our study demonstrates that dancing has benefits on working memory and immediate recall. Prior research indicated that dancing is a multi-faceted exercise that engages individuals' visuospatial skills (de Natale et al., 2017; Douka et al., 2019). One possible explanation is that the nature of dance places a high demand on the spatial cognitive processes of dancers (Douka et al., 2019). Dance may assist older adults in recognizing and managing the distance between themselves and others. Additionally, they must maintain their balance, posture, and movement during the dance (Lazarou et al., 2017). Balance is a crucial functional skill that could influence older adults' daily activities, which could coordinate rhythmic movements by coordinating neurosensory and musculoskeletal systems (de Natale et al., 2017). Moreover, dancing improves flexibility and muscle strength in both the upper and lower limbs, as well as balance and synchronization of movements (Douka et al., 2019). A previous study found that dance is sensitive in enhancing cognitive flexibility in cognitively impaired adults (Wu et al., 2018), and our study showed similar results. The improvisation works of contemporary dance, which is quite of similarity with OSDA, was considered can not only improve the motor act itself but also the way individuals solve specific motor problems when confronted with different external environments and conditions, which is known as cognitive flexibility (Coubard et al., 2011). Our study found that attention was improved after dance intervention, while previous reviews found no effect on attention (de Natale et al., 2017; Hewston et al., 2021). In our meta-analysis, only three studies reported outcomes of attention, and they were assessed using different measurements. Therefore, more studies are required to draw credible conclusions on attention.

Dance activity organically integrates body movements and music, which is a unique non-pharmacological intervention for older adults with MCI. Older adults follow the music and use their body as a carrier to practice dance activities, which can improve not only physical fitness and athletic ability but also improve cognitive function by affecting the microstructure and function of the brain of the patient and improving cardiovascular function.

Previous studies have reported that the fractional anisotropy (FA) value of the left fibrous circuit in older adults with MCI was lower than that of normal people (Zhang et al., 2007), and after 12 weeks of aerobic dance intervention in older adults with MCI, FA values in the bilateral cingulate gyrus, left hippocampus and left superior longitudinal tract, which is closely associated to memory, and cognitive function increased significantly (Zhang et al., 2020). The cingulate tract, superior longitudinal tract, and hippocampus are the most important structures affecting the cognitive function of individuals. The cingulate tract structurally connects the parahippocampal gyrus and the subcallosal region. It functionally plays an important role in the cholinergic system pathway (Peter et al., 2021) and affects the ability of the individual to integrate information, spatial memory, and episodic memory (Chen et al., 2012). The superior longitudinal tract connects the frontal lobe, parietal lobe, occipital lobe, and temporal lobe. It extends through the front and rear of the brain and can transmit audio-visual sensations, thereby directly affecting the executive function and working memory of the individual. The hippocampus is the central structure of the human limbic system and is an important part of the human brain for learning and short-term memory. Dance intervention can promote the FA value of the above three parts, which have irreplaceable importance for the cognition, memory, visuospatial function, and attention of the individual (Bettcher et al., 2016; Zheng et al., 2020). The intensity of dance intervention was reported in some of the included studies (Zhu et al., 2018; Qi et al., 2019; Wu et al., 2019; Bisbe et al., 2020; Fei and Cai, 2020; Zhang et al., 2020; Zhao and Li, 2020; Chang et al., 2021). The level of daily physical activity and self-efficacy of older adults with MCI are lower than those of normal people (Guo et al., 2021). Properly increasing the intensity of physical activity can increase the volume of the atrophied brain region and the cerebral blood volume, thereby improving the spatial memory function of the patient (Maass et al., 2015; Guo et al., 2021). On the other hand, the acceleration of the blood flow rate promotes metabolism (Loprinzi et al., 2019), which supplies sufficient oxygen to the brain. This is beneficial to the production of neurotrophic factors and the generation of blood vessels, synapses, and glia in the brain (Maass et al., 2016) and then increases the spontaneous activity of neurons and the repair capacity of the brain (Deslandes et al., 2009; Zhu, 2018). Moreover, Wang et al. proposed that the improvement of cognition through physical activities can also be associated with the oxygenation of the brain. However, a lack of strong evidence exists (Wang et al., 2021).

In contrast to regular physical exercise, dance activities require participants to complete rhythmic body actions accompanied by music. Therefore, music contributes to the effect of dance activities on the improvement of cognition in older adults. The role of music in the treatment of dementia has already been well-reported. When individuals listen to music, a wide range of parts in the cerebral cortex are involved, which includes auditory, musical syntactic processing, semantics, memory, motor function, and emotional processing (Baird and Samson, 2015). Music can improve the attention, visual-motor coordination, and memory of the individual by activating the frontal cortex of the brain and strengthening neural connections. Therefore, neurological deficits in older adults with MCI can be repaired through music (Thornley et al., 2016; Liu et al., 2019). The included studies applied aerobic dance, square dance, and ballroom dance along with a strong rhythm. Sound waves can resonate with the tissues and cells in the human body. When the excitability of cells increases, the content of brain-derived neurotrophic factor (BDNF) increases significantly, which improves the advanced functions of the human brain (Griser et al., 2016). Dancing to music can help elderly individuals release neurotransmitters, such as norepinephrine and acetylcholine, and repair the defective neural network and neural circuit functions, which thereby improves global cognition and its various sub-domains (Chang et al., 2015; Yin et al., 2018). Therefore, both body movement and music in dance could be presumed reasonably to promote the release of neurotrophic factors and increase neural activity by affecting the function and structure of the brain areas of the individuals. The precise, dual support of physical activity and music that dance activities have a significant effect on the cognitive function of older adults with MCI.

However, as only two of the included studies in our meta-analysis gathered follow-up data, there was a lack of information to demonstrate the maintenance of the intervention effect. According to Jia et al., the recurrence rate of the experimental group was significantly lower than that of the control group (Jia, 2017). And Zhu et al. followed up with patients for another 3 months after the dance intervention was completed. The follow-up data illustrated that the previously improved memory was attenuated (Zhu et al., 2018).

### Subgroup analysis

We conducted subgroup analysis based on several variables, including the type, frequency, and length of the intervention.

Through the intervention implementation content reported in the original studies, studies were divided into OSDA and CSDA. CSDA had strong predictability and was characterized by less varied movements and a relatively single and stable form, whereas OSDA had relatively strong unpredictability and uncertainty and was flexible, irregular, and more diverse. The results of this study showed that even though the effects of both dance modalities were positive and significant, the effects of OSDA were significantly better than that of CSDA (*p* = 0.0002).

Both OSDA and CSDA at certain intensities can have some positive effects on the cognitive function and flexibility of the individual (Dai et al., 2013). The effects of the two different motor skills of dance have some differences. Previous studies have reported that complex environments provided by open-skill activities can increase the thickness of the brain cortex in rodents (Nithianantharajah and Hannan, 2006), increase the number of nerves (Artola et al., 2006), and strengthen the transmission function of synapses (Van Praag et al., 2000). The gray matter volume of the prefrontal cortex, temporal gyrus, frontal lobe, parietal lobe, and occipital lobe of participants involved in OSDA was significantly larger than that of the control group, and the increase in gray matter volume in the areas mentioned above was closely associated with motor execution, learning, working memory, and visual processes (Jacini et al., 2009). Therefore, OSDA had a better effect on cognitive function improvement than CSDA (Gu et al., 2019). In a healthy elderly population, OSDA had significant effects on visual-auditory perception, visuospatial function (including visuospatial attention and visuospatial working memory) (Guo et al., 2016; Tsai et al., 2016), and attentional inhibition control (Tsai et al., 2016), and was more effective than CSDA on cognitive flexibility (Dai et al., 2013), whereas CSDA only had a significant effect on working memory (O'Brien et al., 2017; Tsai et al., 2017). The above results were also reported in a one-shot exercise study (O'Brien et al., 2017). Individuals have the visuospatial capacity for active manipulation and passive storage, with active manipulation showing signs of aging earlier than passive storage capacity. Researchers have suggested that the decline of active manipulation ability caused by aging is irreversible, and both OSDA and CSDA have no significant effect on it. However, passive storage capacity is more susceptible to OSDA (Vecchi et al., 2005), which is consistent with the results from previous studies (Erickson et al., 2011). Exercising in a cognitively challenging environment can produce better results for the combined effects of intrinsic and explicit cognition, induces neurological and cognitive benefits, and then achieves better cognitive benefits (Fabel et al., 2009; Fan and Wang, 2009).

Based on the results of this study, we found that intervention outcomes were significantly effective regardless of the length of intervention. However, the longer the intervention length was, the better effect it had. One possible explanation is that, unlike exercise intervention (Cai et al., 2022), older adults participating in dance activities are first required to learn and be familiar with the dance movements before they can master the correct movement patterns. Therefore, the early effect of dance intervention can be lower than that of exercise intervention. After some time, the familiarity of the older adults with the movement pattern improved, and the effect of promoting cognitive function gradually appeared. Therefore, within a certain range, a longer intervention length may also be one of the reasons for the better intervention effect. However, there was no clear consensus on the dose and type of intervention to improve cognitive functioning in older adults with cognitive impairment. In terms of intervention frequency, the results showed that the effect of 1–2 times per week was significant and that of 3 times per week was not significantly effective. We speculate that the energy and physical strength of older adults are relatively limited; therefore, interventions with moderate frequency may be more effective than those with high frequency. However, this lacks strong evidence to support it.

Although no significant difference was found between the two age groups ( ≤ 70 years old and >70 years old) using MMSE or MoCA, the effects in group 1 were greater than those in group 2 (Table 6), indicating that dance intervention may be more effective in younger older adults. Previous research found that increasing age is an independent risk factor for MCI; however, there is a trend that MCI is becoming more common in younger (Qiu et al., 2018) people. According to the findings regarding age, earlier dance intervention in older adults with MCI may be more effective.

When estimating effect size, high heterogeneity was discovered, which could be attributed to differences in design between the included studies. As previously stated, the characteristics of the included studies differ. Ten RCTs and two CTs were included in our study. The use of randomization may aid in providing stronger evidence. There are also various intervention types, such as OSDA (choreographed exercise and ballroom dance) and CSDA (square dance and aerobic dance). Different types of interventions stimulate individuals' brains in different ways. The time, frequency, and length of the interventions in the trials were clearly different, which may be one of the most important reasons for the heterogeneity. Because the best dance intervention dose is still unknown, determining appropriate intervention doses for older adults with MCI is difficult. Because the study designs of the included trails are based on a thorough consideration of the subjects' actual situation and physical condition, there are some differences in the effect.

The findings above should be interpreted with caution. As in the above results, while a high degree of heterogeneity could be seen in MMSE, MoCA, balance, and CDSA, Egger's asymmetry test indicates a symmetrical distribution of effect sizes and a low risk of publication bias.

### Limitation

First, to ensure the quality of the included studies, all the included studies were written in Chinese or English and published in the core journals of China and abroad. Papers, conference papers, gray literature, unpublished studies, and studies written in other languages were not included in this meta-analysis, which can lead to publication bias and introduce language bias, as it may not cover all potential studies. Second, using MMSE or MoCA alone to evaluate global cognition should not be regarded as sufficient as a screening tool as they cannot substitute for neuropsychological testing. Third, some heterogeneity was found among the studies, and subgroup analysis did not reduce the heterogeneity. Many methodological weaknesses, such as incomplete reports of participants' gender and the use of blinding, were identified, which can question the validity of the results. Older adults who were identified as MCI patients in the original study were eligible to be included in this meta-analysis. Therefore, the conditions of the subjects, the research design, and the implementation can all be sources of heterogeneity, and certain interactive effects can be found among the above factors. Furthermore, regarding missing data, three studies used an intention-to-treat analysis (Zhu et al., 2018; Wu et al., 2019; Wang et al., 2020), and seven studies used a per-protocol analysis (Doi et al., 2017; Lazarou et al., 2017; Qi et al., 2019; Bisbe et al., 2020; Zhang et al., 2020; Zhao and Li, 2020; Chang et al., 2021), while two studies had no missing data (Jia, 2017; Fei and Cai, 2020). Different ways of dealing with missing data could result in different degrees of bias. This should be noticed and considered while making conclusions.

### Implications for practice and research

First, at present, in the dance activity intervention for older adults with MCI, the specific dance types, frequency and length of intervention, and measurements of outcome indicators were many, which can affect the authenticity and reliability of the research results to a certain extent. Therefore, to improve the methodological quality, more randomized controlled trials with rigorous study designs and larger sample sizes are required to strengthen the control of bias from the aspects of the use of blinding and allocation concealment. Second, the effects of different types of dance activities and the different types and doses of intervention should be compared to determine better and more reasonable content and extent of dance activity intervention. Third, to further improve the in-depth discussion of dance activity intervention, future research should involve the analysis of brain structure, function, cardiovascular function, and their underlying mechanisms. Furthermore, an intention-to-treat analysis is suggested when dealing with missing data, as it is considered better to avoid bias.

## Conclusion

Dance activities have a positive effect on the cognitive function, memory, visuospatial function, cognitive flexibility, attention, and balance of older adults with MCI. However, because of the evidence base, the results should be interpreted with caution. Nevertheless, dance activity can play an important role in improving the cognitive function of elderly people as a basis for a physically active lifestyle, and they can get a variety of physiological, social, and cognitive health benefits. Both body movement and music elements in dance could be presumed reasonably to promote the cognition of older adults with MCI. Future high-quality research is required to establish certainty in the effectiveness of dance activity in older adults with MCI by searching for optimal programs, dose-response relations, and long-term sustainability.

## Data availability statement

The original contributions presented in the study are included in the article/supplementary material, further inquiries can be directed to the corresponding author.

## Author contributions

YY and XL contributed to the conception of the study. YY and WL contributed significantly to the literature search. YY performed the data analysis and wrote the manuscript. XL helped perform the analysis with constructive discussions. All authors read and approved the final manuscript.

## Conflict of interest

The authors declare that the research was conducted in the absence of any commercial or financial relationships that could be construed as a potential conflict of interest.

## Publisher's note

All claims expressed in this article are solely those of the authors and do not necessarily represent those of their affiliated organizations, or those of the publisher, the editors and the reviewers. Any product that may be evaluated in this article, or claim that may be made by its manufacturer, is not guaranteed or endorsed by the publisher.
